# Within-Day Dynamics of Self-Efficacy and Smoking Attitudes: Ecological Momentary Assessment Study of Motivation to Quit and Forgoing Behavior

**DOI:** 10.2196/87117

**Published:** 2026-07-23

**Authors:** Amanda L Rebar, Liyan Xiong, Chih-Hsiang Yang, Dèsirée Vidaña-Pérez, James Hardin, Minji Kim, James F Thrasher

**Affiliations:** 1 Department of Health Education, Promotion, & Behavior, Arnold School of Public Health, University of South Carolina, Discovery 1, 515, 915 Greene Street, Columbia, SC, 29208-4001, United States, 1 803 777 7603; 2 Department of Epidemiology & Biostatistics, Arnold School of Public Health, University of South Carolina, Columbia, SC, United States; 3 Department of Exercise Science, Arnold School of Public Health, University of South Carolina, Columbia, SC, United States

**Keywords:** smoking cessation, just-in-time adaptive intervention, dynamics, health behavior, self-efficacy

## Abstract

**Background:**

Motivation to quit smoking and decisions to smoke or forgo smoking vary throughout the day. However, little is known about how within-day patterns of psychological states such as self-efficacy and attitudes toward smoking relate to these determinants of smoking cessation attempts. Identifying these dynamic processes can inform the development of more precisely timed and tailored digital interventions.

**Objective:**

This study aimed to identify distinct within-day trajectories of self-efficacy for cutting down on cigarettes smoked and attitudes toward smoking, and to examine how these trajectories predicted end-of-day motivation to quit and same-day cigarette forgoing (ie, choosing not to smoke cigarettes that one would normally smoke).

**Methods:**

People who smoked at least 10 cigarettes a day at baseline (N=348, mean age 44.6, SD 12.1 years; n=212, 60.9% female) received smartphone surveys about 4‐5 times a day after logging each cigarette, producing 15,614 surveys over 2561 days. Trajectories of self-efficacy and smoking attitudes were modeled at the person-day level using smooth functions, and 6 daily parameters of change (overall level, range of change, volatility, overall trend, acceleration of change, and trajectory shape [trend×acceleration]) were extracted. These parameters were then entered as predictors of (1) end-of-day motivation to quit (linear mixed models) and (2) whether participants forwent cigarettes that day (binomial generalized linear mixed models).

**Results:**

Higher overall self-efficacy consistently predicted both greater end-of-day motivation and greater odds of forgoing. Upward trends and acceleration in self-efficacy further predicted greater odds of forgoing, indicating that days when confidence not only increased but did so quicker were most strongly associated with forgoing cigarettes that day. Less favorable attitudes toward smoking predicted greater motivation to quit and increased likelihood of forgoing cigarettes. Broader ranges of daily change in attitudes were linked with stronger motivation to quit and greater odds of forgoing, while more moment-to-moment volatility was associated with reduced odds of forgoing cigarettes that day.

**Conclusions:**

Dynamic features of self-efficacy and smoking attitudes, such as overall level, trend, and acceleration, were robust predictors of daily motivation to quit and cigarette forgoing. These findings highlight that the way self-efficacy and attitudes shift across the day is meaningful beyond their overall levels. Just-in-time adaptive interventions may be more effective if they monitor and respond to varying trajectory features rather than focusing on static states, supporting a shift toward dynamically aware intervention strategies in digital health.

## Introduction

### Background

Smoking cigarettes severely impacts the quality and longevity of a person’s life [1]. Tobacco causes systemic harms on the body and mind, leading to a wide assortment of diseases, including cancers, heart disease, lung diseases, and type 2 diabetes [1], that result in the premature deaths of more than 8 million people each year globally [2]. As the most common method of tobacco consumption, smoking cigarettes remains the primary target of most public health measures to prevent tobacco-related deaths [3]. Although not all harms of smoking are reversible, quitting before the age of 40 years can reduce early mortality risk by about 90% [4]. To reduce the massive burden of smoking, there is a need to better understand the dynamics of modifiable predictors of motivation to quit and cessation behaviors.

Motivation to quit smoking plays a pivotal role in initiating quit attempts and is a predictor of eventual cessation success [5,6]. When a person reports motivation to quit smoking, it reflects their readiness to change and serves as a crucial psychological trigger that shifts them from contemplation to action [7,8]. Research consistently shows that people with stronger motivation to quit smoking are significantly more likely to initiate serious quit attempts. For example, estimates from the International Tobacco Control Four Country Survey found that people who smoke and reported strong motivation to quit were more than twice as likely to attempt quitting within 6 months compared with those with low motivation [5,9], and a large longitudinal study of US people who smoke showed that those expressing strong motivation to quit had over a 2.5-fold increased likelihood of making a quit attempt [8].

Successful smoking cessation is influenced not only by a person’s motivation to quit but also by engagement in specific preparatory behaviors (sometimes referred to as microbehaviors), such as stubbing out or forgoing smoking cigarettes in situations when one craves a cigarette or would normally smoke [10]. Forgoing cigarettes can signal readiness to quit and consistently and independently predicts subsequent quit attempts [11-14], although the evidence is mixed on whether forgoing leads to successful cessation, which is primarily determined by one’s level of nicotine dependence [9,10,15]. Nevertheless, forgoing cigarettes moves people toward cessation attempts, even if they are not motivated to quit, and, as such, could serve as an intervention target to promote, reinforce, and expand upon.

Motivation to quit smoking and microbehaviors of cessation, such as forgoing cigarettes, are reliably predicted by a person’s attitudes toward smoking and their beliefs that they can cut down on smoking (ie, self-efficacy) [15-17]. Positive attitudes about smoking indicate a pleasant valuation of smoking and anticipated pleasure from the act, which can have a strong hedonic motivational sway on behavior [18,19], making it less likely that a person will be motivated to quit or forgo smoking. Alternatively, when people have strong self-efficacy to cut down on smoking, they enjoy an increased likelihood to overcome barriers and maintain control over smoking urges [20]. This self-confidence increases the likelihood of being motivated to quit smoking and engaging in cessation-related microbehaviors such as forgoing cigarettes when tempted [17]. Meta-analytic findings show that the association between self-efficacy and quit attempts or short-term abstinence is moderate to strong, with effect sizes typically ranging from *r*=0.30 to 0.50 [17]. Attitudes similarly demonstrate moderate effects on motivation and behavior, often in the range of *r*=0.20-0.40 [15]. These effects highlight that, while neither factor alone guarantees quitting, their combined influence plays a substantial role in driving cessation-related motivation and behavior.

For decades, public health efforts have promoted smoking cessation through persuasive campaigns aimed at motivating people to quit smoking. Although these campaigns effectively raise awareness about the harms of smoking and can be effective in promoting cessation [21,22], for many people who smoke, campaigns often fall short in translating awareness and cessation attempts into sustained cessation [23,24]. Despite the widespread knowledge of the harms of smoking [4], more than 1 in 5 adults around the world still smoke cigarettes regularly [3]. One reason that these static intervention approaches have limited effectiveness is that smoking behaviors and the motivational factors that influence them fluctuate throughout the day, changing in line with a person’s affective and cognitive states, external demands, responsibilities, environment, and social contexts [25,26]. Prior work has documented these momentary fluctuations but has largely focused on levels of experience or discrete events. Less is known about how these processes unfold over time within a day, including patterns of change such as whether they improve or worsen (ie, direction), how much they fluctuate (ie, variability), and how patterns of change unfold over time (ie, dynamics). To be more effective, smoking cessation interventions may need to align not only with the presence of these fluctuations but also with their underlying temporal patterns [27,28].

### The Present Study

This study examined within-day dynamics of 2 key constructs relevant to smoking behavior: attitudes toward smoking (ie, momentary evaluations of smoking as good or bad) and self-efficacy for forgoing cigarettes (ie, confidence in one’s ability to resist smoking in the moment). We focused on how these constructs change across the course of a day, including their levels and patterns of change. We addressed 2 primary research questions: (1) What distinct within-day trajectory patterns characterize changes in attitudes toward smoking and self-efficacy for forgoing cigarettes? and (2) Are features of these trajectories (eg, overall level, variability, and direction of change) associated with end-of-day motivation to quit smoking and the likelihood of forgoing a cigarette on that day?

## Methods

### Study Procedures

Data were from a longitudinal clinical trial using ecological momentary assessment (EMA) [29-31]. Recruitment occurred in central New York and central South Carolina from June 2019 to June 2021, with interruptions during the initial COVID-19 outbreak and closures [29]. Prior to the pandemic, the New York site recruited locally at smoke shops in 6 urban and rural areas selected to capture areas of low household income. Research staff stationed outside the shops invited adults to participate, screened interested individuals for eligibility, and, if eligible, provided participants with study materials and baseline measures on site. In South Carolina, recruitment was conducted through online advertisements and flyers that directed interested individuals to a web-based eligibility screener. Adults (ie, those older than 18 years) were eligible to participate if they had smoked at least 100 cigarettes in their lifetime and at least 10 cigarettes a day in the prior month, with smoking confirmed via exhaled CO (Covita) of at least 8 ppm. Eligible respondents were then contacted to confirm eligibility and schedule an in-person orientation and materials pickup. After the onset of COVID-19, both sites shifted to recruitment through social media advertisements, orientations were conducted virtually, and study materials were provided during scheduled in-person visits to a university or other central location.

For the intervention, participants attended a 45-minute orientation session and were provided with a 14-day supply of their preferred cigarette brand, with cigarette packs modified to reflect their randomized labeling condition: control (standard text-only health warnings), insert-only (packs containing paper inserts with efficacy messages), graphic-only (packs displaying pictorial health warnings), or insert plus graphic (packs including both pictorial warnings and efficacy message inserts). Data from all participants across the 4 labeling conditions were included in the present analyses, with study condition statistically controlled in all models.

Across all conditions, participants logged each cigarette smoked using the study app. Additionally, participants completed a 1‐ to 3-minute survey following approximately 4 or 5 of the logged cigarettes (randomly sampled each day, with sampling frequency matched to baseline smoking rate), as well as following the first cigarette from each new pack. Data from all participants across the 4 labeling conditions were included in the present analyses, with study condition statistically controlled in all models. Omitting data from days with fewer than 4 smoking survey completions resulted in exclusion of 3429 assessments (18%) for 363 days from 344 participants. This resulted in data of 15,614 assessments of 2561 individual days from 348 individuals for this study.

### Ethical Considerations

The study was approved by the University of South Carolina Institutional Review Board (Pro00083728). Written informed consent was obtained from all participants after eligibility screening. Participant confidentiality was protected by separating identifying information from study data and assigning each participant a unique study ID. Participants received a 2-week supply of their preferred cigarette packs modified with the randomized warning labels and an additional US $150 upon completion of the study.

### Measures

#### Demographic Characteristics

Participants self-reported their age (in years), sex at birth (male and female), race or ethnicity (White/Black or African American, Latino/Latina/Latinx, Asian, Native Hawaiian or Pacific Islander, American Indian or Alaska Native, and Not Listed [Please Specify]), educational attainment (grade school or some high school; completed high school; technical/trade school or community college; some university, no degree; completed university degree; and postgraduate degree), and annual household income (<US $10,000; US $10‐000-US $29,999; US $30,000-US $44,999; US $45,000-US $59,999; US $60,000-US $74,999; US $75,000-US $99,999; US $100,000-US $149,999; US $150,000 and over; and Prefer not to answer) at study baseline.

#### Momentary Self-Efficacy of Cutting Down on Cigarettes Smoked

During each assessment, participants reported how easy it would be to cut down on the number of cigarettes smoked at that moment using the response scale: 1 (Not at all) to 7 (Extremely).

#### Momentary Attitudes of Smoking

Participants responded to the question, “Right now, you feel like smoking is?” using the response scale: 1 (Bad) to 7 (Good) [11,12] at every assessment.

#### Momentary Motivation for Quitting Smoking

Participants responded to the question, “How motivated are you to quit smoking?” using the response scale: 1 (Not at all) to 7 (Extremely). Given the stability of this measure within-day (intraclass correlation 0.80, 95% CI 0.78‐0.82) and our focus on motivation as an outcome, we used only the final response from each day, recording the survey time as a covariate in the models (see Data Management and Analyses section) [11,12].

#### Daily Forgoing

As part of the end-of-day survey (administered between 7 PM and midnight, with a reminder at 9 PM if not yet completed), participants reported whether they had forgone any cigarettes they normally would have smoked in the past 24 hours (yes/no).

### Data Management and Analyses

All analyses were conducted in R (version 4.4.1; R Core Team) [32]. To enable meaningful analysis of change over time, only days with at least 4 or more assessments were used for the trajectory cluster analyses [33]. To better reflect participants’ daily patterns, the study day was defined as starting at 4 AM instead of the standard midnight start.

Trajectory cluster analysis (using the *R* package *traj* [34]) provides insights into patterns of longitudinal change based on calculated parameters of change [33]. The parameters of change were 19 measures of longitudinal change for each day spanning measures of overall values (eg, mean), variation over time (eg, SD, range), direction of change (eg, slope of linear model), and fluctuation and nonlinearity (eg, maximum of the first and second derivatives) [34]. Only those parameters that were not correlated at *r*=0.95 or higher were used in the principal factor analysis with varimax rotation and k-means cluster analysis. The number of clusters was determined based on inspection of the Euclidean distance and Friedman Index values across potential solutions along with interpretability of the cluster solutions [35,36]. To examine whether specific features of motivational trajectories predicted daily likelihood of forgoing cigarettes and end-of-day motivation to quit, we included continuous trajectory features (mean level, variability, and rate of change in momentary motivation) as predictors in binomial and linear mixed-effects models, respectively [37]. All models included the covariates of age, sex at birth, race or ethnicity, education, income, and intervention condition.

To further evaluate whether associations between trajectory features were codependent, we tested moderation by creating interaction terms between mean-centered change parameters that were conceptually related (eg, trajectory shape, defined as the product of mean-centered overall trend and acceleration of change). For all models with end-of-day motivation as the outcome, we included time of day (in numeric 0‐24 hours) the final survey was completed as a covariate. All continuous predictors were mean-centered and scaled prior to estimation to reduce multicollinearity and improve the interpretability of model coefficients. Model diagnostics included checks of residual normality, heteroscedasticity, and multicollinearity (variance inflation factors), which all indicated acceptable model fit.

## Results

### Sample Characteristics

Participants (N=348) were, on average, aged 44.6 (SD 12.1) years, ranging from 20 to 72 years. Most were female (212/348, 60.9%), with 37.9% (132/348) identifying as male, and 1.1% (4/348) participants not reporting their gender. Most participants were White (282/348, 81.0%) and had education beyond a high school diploma (205/348, 58.9%), including 43.0% with some college or technical training and 16.6% with a university or graduate degree. Most participants reported annual household incomes below US $60,000, with the largest groups earning US $10,000‐US $29,999 (27.6%) or US $30,000‐US $44,999 (19.8%), and smaller proportions in higher income ranges (6% at US $100,000 or above).

### Smoking Survey Completion Descriptives

Multilevel binomial modeling [37] was used to test for differences between excluded and included data. Days that were excluded (ie, those with fewer than 4 smoking surveys completed) had slightly higher self-efficacy for cutting down on smoking (odds ratio [OR] 1.06, 95% CI 1.01-1.12), stronger motivation to quit smoking (OR 1.10, 95% CI 1.05-1.16), and higher probabilities of forgoing smoking (OR 1.66, 95% CI 1.47-1.87) than days that were included in analyses. There were no differences in smoking attitudes between excluded and included days (OR 0.97, 95% CI 0.93-1.01).

In person-level analyses across the full study period, higher mean self-efficacy (*b*=0.05, SE=0.02; *P*<.001), more negative mean attitudes toward smoking (*b*=−0.04, SE=0.01; *P*<.001), and higher mean motivation (*b*=0.06, SE=0.01; *P*<.001) were each associated with a greater proportion of forgoing cigarettes across the study period. The overall model explained 18.1% of the variance in forgoing behavior (*R*²=0.18). Multilevel linear modeling [37] was used to test for differences between participants with more versus less excluded days across these relations. Participants with a higher proportion of excluded days showed a modestly higher proportion of forgoing cigarettes overall (*b*=0.96, SE=0.43; *P*=.03); however, interactions between proportion excluded and mean self-efficacy for cutting down on smoking (*b*=0.04, SE=0.08; *P*=.65), mean attitudes toward smoking (*b*=−0.08, SE=0.06; *P*=.18), and mean motivation to quit smoking (*b*=−0.10, SE=0.07; *P*=.18) were not significant, indicating that these associations were consistent regardless of exclusion status.

Participants most frequently completed smoking surveys in the late afternoon and early evening, with the highest proportions between 4 PM and 6 PM (11.9%) and between 6 PM and 8 PM (12.4%). Fewer surveys were completed overnight, especially between 2 AM and 4 AM (1.5%). Overall, survey completions increased from the early morning, peaked in the evening, and declined through the night: early morning (4 AM to 10 AM)=19.6%, midday (10 AM to 4 PM)=33.9%, evening (4 PM to 10 PM)=35.1%, and night (10 PM to 4 AM)=11.4%.

### Cluster Trajectory Analyses

#### Overview

Analyses supported 5-factor solutions for the trajectory clusters of momentary self-efficacy and attitudes. As shown in Table 1, 5 components together accounted for a substantial proportion of the total variance in both momentary self-efficacy and attitudes toward smoking (88% and 87%, respectively). Although the first 3 components explained the largest portions of variance, the fourth and fifth components each contributed an additional 9%‐16%, indicating that meaningful structure remained beyond a 3-factor model. The 5-factor solution provided a more comprehensive representation of the patterns observed across the trajectories. Cluster fit indices were consistent with this interpretation: Euclidean distances decreased substantially from 3- to 5-cluster solutions, indicating improved model compactness, while silhouette coefficients remained small and stable, suggesting that the additional clusters captured subtle but interpretable distinctions rather than noise. The retention of 5 distinct trajectory components best represents systematic heterogeneity in motivational change over time for both variables.

**Table 1.** Factor loadings and variance explained for the 5 retained components for trajectory clusters of momentary motivation variables.

Factors	1	2	3	4	5
Momentary self-efficacy for cutting down on smoking cigarettes					
SS loadings^a^	5.94	2.43	2.83	1.55	2.82
Proportion of variance explained	0.33	0.14	0.16	0.09	0.16
Euclidean distance	15,345	11,386	11,219	7020	7552
Silhouette coefficients	N/A^b^	0.17	0.05	0.04	−0.00
Momentary attitudes toward smoking					
SS loadings	6.26	2.78	2.44	1.55	2.54
Proportion of variance explained	0.35	0.15	0.14	0.09	0.14
Euclidean distance	15,345	11,700	8328	8361	7736
Silhouette coefficients	N/A^b^	0.18	0.12	0.02	0.01

a Squared multiple correlation coefficients (loadings).

b Not applicable.

#### Self-Efficacy for Cutting Down on Cigarettes

The model parameters contributing most to this explained variance were range of change (ie, maximum – minimum values), overall level (ie, mean self-efficacy per day), volatility (ie, rate of intersection with the mean), overall trend (ie, mean of the first derivative), and acceleration of change (ie, mean of the second derivative). The intraclass correlations revealed that the cluster-derived parameters of change in self-efficacy varied across days. Overall self-efficacy level was most consistent, followed by range of change and volatility, whereas trend and acceleration were least stable, suggesting that both the direction and the pace of change in self-efficacy to cut down on cigarettes shifted considerably from day to day within the same person.

**Figure 1.**
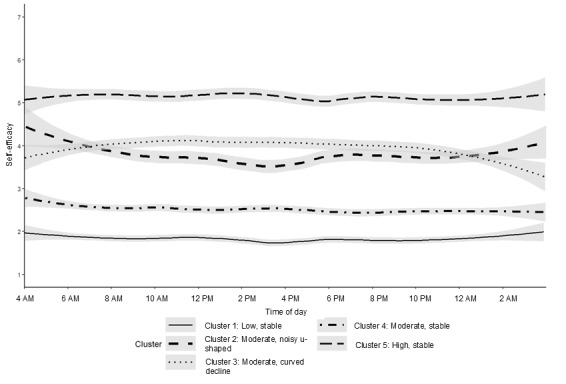
Daily trajectories of self-efficacy for cutting down smoking by cluster. Smoothed median momentary self-efficacy scores are shown across a 24-hour period beginning at 4 AM. Shaded areas represent 95% CIs..

The parameters of change for each cluster are shown in Table 2, and Figure 1 shows the point median of the smoothed daily trajectory for each cluster. The first cluster, comprising 21.6% of days, represents a “low, stable” self-efficacy trajectory. These days are characterized by a low range of change and minimal volatility, reflecting consistently low and unchanging self-efficacy across the day. Additionally, both the overall trend and the acceleration of change are 0, reinforcing that there is no meaningful trend or acceleration: self-efficacy remains flat throughout the day.

The second cluster, representing 18.0% of days, represents a “moderate, noisy U-shaped” self-efficacy trajectory. This trajectory reflects a midrange level of self-efficacy (3.61 out of the 1‐7 scale). The range of change (1.63) indicates moderate to large within-day variation with frequent, irregular ups and downs across the day. The relatively high volatility (0.63) suggests many moment-to-moment deviations around a person’s daily average. The overall trend is nearly flat (–0.02), but the positive acceleration (0.39) indicates an upward-curving pattern, suggesting that initial dips in self-efficacy are followed by rebounds later in the day.

The third cluster, representing 25.4% of days, reflects a “moderate, curved declined” self-efficacy trajectory. This trajectory reflects a moderate overall level of self-efficacy (4.04) with broad, systematic swings across the day. The range of changes is relatively large (2.16), reflective of big differences in self-efficacy at some points throughout the day. Volatility is modest (0.46), suggesting some random moment-to-moment deviations, but the negative acceleration (–0.24) points to a structured pattern in which initial increases are followed by sharp declines later in the day.

Cluster 4 (23.8% of days) represents a “moderate, stable” self-efficacy trajectory. The average daily level is 2.49 on the 1‐7 scale, indicating a moderate sense of confidence in cutting down on cigarettes. The range of change is 1.44, reflecting moderate within-day changes in self-efficacy. Volatility is 0.35, suggesting occasional crossing above and below the daily average but with less frequent fluctuations than other clusters. The overall trend is slightly negative (–0.02), indicating a mild decline over the day, while the acceleration of change is near zero (0.01), consistent with a steady pattern without substantial speeding up or slowing down of change.

Cluster 5 (11.3% of days) reflects a “high, stable” self-efficacy trajectory. The average daily level is 5.15, the highest among all clusters, suggesting strong confidence throughout the day in cutting down on cigarettes. The range of change is very low at 0.26, indicating very little variation in self-efficacy across the day. Volatility is also minimal (0.07), consistent with near-constant self-efficacy levels. The overall trend and acceleration of change are both near zero, reflecting a flat, stable trajectory without meaningful increases or decreases over time.

**Table 2.** Average parameters of change and proportion of days represented by each daily self-efficacy trajectory cluster.

Cluster	ICC^a^	Cluster 1	Cluster 2	Cluster 3	Cluster 4	Cluster 5
Cluster name	ICC% (95% CI)	Low, stable	Moderate, noisy U-shaped	Moderate, curved decline	Moderate, stable	High, stable
Momentary self-efficacy for cutting down on smoking cigarettes, n (%)	N/A^b^	552 (21.6)	460 (18.0)	651 (25.4)	609 (23.8)	289 (11.3)
Range of change^c^	43.5 (39.8‐47.4)	0.00	1.63	2.16	1.44	0.26
Overall level^d^	89.9 (88.5‐91.2)	1.86	3.61	4.04	2.49	5.15
Volatility^e^	37.0 (33.6‐40.8)	0.00	0.63	0.46	0.35	0.07
Overall trend^f^	15.6 (13.5‐18.0)	−0.00	−0.02	−0.02	−0.02	0.00
Acceleration of change^g^	15.2 (13.2‐17.6)	0.00	0.39	−0.24	0.01	0.00

a ICC: intraclass correlation.

b Not applicable.

c Range of change: Maximum – minimum.

d Overall level: mean of scores per person.

e Volatility: rate of intersection with the mean.

f Overall trend: mean of the first derivative.

g Acceleration of change: mean of the second derivative.

#### Attitudes Toward Smoking

Most variance was explained by the same change parameters as for the trajectory cluster analyses of self-efficacy. The cluster-derived parameters of change in attitudes toward smoking also varied in their stability across multiple days: people’s overall attitude levels were most consistent, followed by the range of change and volatility throughout the day, whereas trend and acceleration were least stable, indicating that the direction and pace of attitude change shifted day-to-day within the same person.

**Figure 2.**
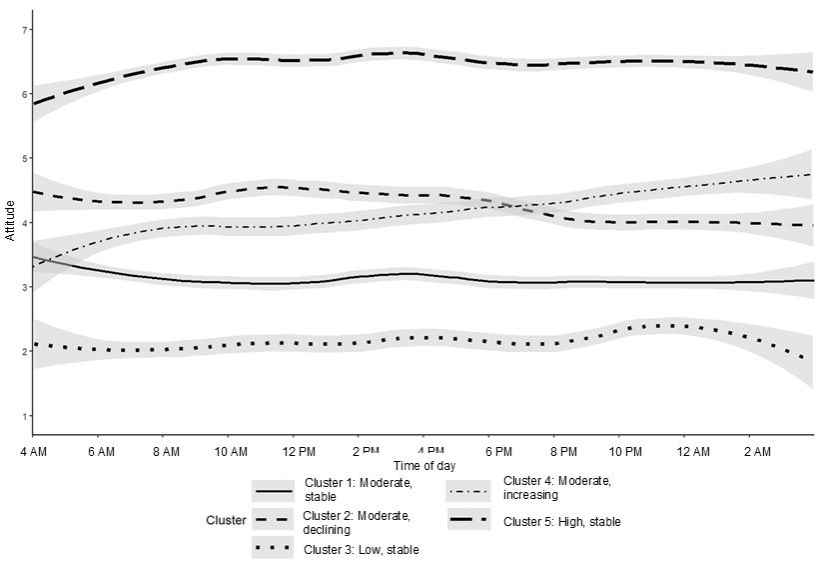
Daily trajectories of attitudes toward smoking by cluster. Smoothed median values of momentary attitude scores are plotted across a 24-hour period starting at 4 AM. Clusters capture distinct within-day patterns of attitude fluctuations. Shaded areas around each line indicate 95% CIs.

The parameters of change for each cluster are shown in Table 3, and Figure 2 shows the point median of the smoothed daily trajectory for each cluster. Cluster 1 (27.8% of days) represents a “moderate, stable” trajectory of attitudes toward smoking. This cluster is characterized by a moderate overall attitude toward smoking, with an average daily score of 3.09. Attitudes show modest change within the day (range of change: 1.16) and some variability around the daily average (volatility: 0.41). The overall trend and acceleration are near zero, indicating generally stable attitudes without meaningful increases or decreases throughout the day.

Cluster 2 represents days with a “moderate, declining” trajectory of attitudes toward smoking, representing 27.7% of days. Days in this cluster exhibit a moderate average attitude level (4.34) but with relatively more range of change (2.21) and volatility (0.53). The overall trend is notably negative (−0.18), suggesting that attitudes tend to decline over the course of the day, although the negative acceleration (−0.23) indicates that this decline may decelerate or level off later in the day.

Cluster 3, representative of 17.1% of days, reflects a trajectory of “weak, stable” attitudes toward smoking. This cluster reflects consistently unfavorable attitudes toward smoking, with a low overall average (2.28) and range of change (0.04) and volatility (0.01) across the day. The trend and acceleration values are near zero, indicating stable attitudes that do not meaningfully change within these days.

Cluster 4 represents 16.8% of days, representative of “moderate, increasing” trajectories of attitudes toward smoking. Attitudes in this cluster average moderately high (4.03) and show relatively high range of change (2.22) and volatility (0.55) within-day. The positive overall trend (0.21) suggests that attitudes toward smoking tend to become more positive throughout the day, and the positive acceleration (0.36) indicates that this increase may be accelerating as the day progresses.

Cluster 5, representative of only 10.7% of days, reflects a “strong, stable” trajectory of attitudes toward smoking. This cluster is defined by an overall positive average attitude toward smoking (6.36) with very limited range of change (0.17) and low volatility (0.04) across the day. The near-zero overall trend and acceleration indicate that attitudes remain consistently positive and stable without meaningful change throughout the day.

**Table 3.** Average parameters of change and proportion of days represented by each daily attitude trajectory cluster.

Cluster	ICC^a^	Cluster 1	Cluster 2	Cluster 3	Cluster 4	Cluster 5
Cluster name	ICC% (95% CI)	Moderate, stable	Moderate, declining	Weak, stable	Moderate, increasing	Strong, stable
Momentary attitudes toward smoking, n (%)	N/A^b^	712 (27.8)	710 (27.7)	437 (17.1)	429 (16.8)	273 (10.7)
Range of change^c^	48.8 (45.1‐52.8)	1.16	2.21	0.04	2.22	0.17
Overall level^d^	90.2 (88.8‐91.5)	3.09	4.34	2.28	4.03	6.36
Volatility^e^	37.0 (33.6‐40.09)	0.41	0.53	0.01	0.55	0.04
Overall trend^f^	15.2 (13.2‐17.6)	−0.01	−0.18	−0.00	0.21	0.01
Acceleration of change^g^	16.3 (14.2‐18.8)	0.00	−0.23	0.01	0.36	−0.01

a ICC: intraclass correlation.

b Not applicable.

c Range of change: Maximum – minimum.

d Overall level: mean of scores per person.

e Volatility: rate of intersection with the mean.

f Overall trend: mean of the first derivative.

g Acceleration of change: mean of the second derivative.

### Features of Trajectory Predicting End-of-Day Motivation to Quit

The models presented in Table 4 show that change features of within-day self-efficacy trajectories predicted motivation to quit at the last cigarette smoked each day. Overall level of self-efficacy was the strongest predictor of end-of-day motivation, indicating that on days when people generally felt more confident about cutting down, they also tended to feel motivated to quit at the end of the day. Volatility was not a significant predictor, suggesting that the degree to which self-efficacy fluctuated across the day did not significantly impact whether people felt motivated to quit at the end of the day or not. Both overall trend and acceleration of change were positively associated with motivation to quit, showing that when self-efficacy moved consistently upward or downward and when those changes became sharper over time, people reported higher motivation to quit at the end of the day. In contrast, the range of change across the day and the combined trajectory shape were not significant predictors, indicating that the sheer size of daily swings or the interplay of slope and curvature of how self-efficacy changed throughout the day did not reliably relate to motivation to quit. Finally, survey timing mattered: motivation to quit was slightly lower when the end-of-day report was completed later in the evening. Random effects further showed that days differed in typical levels of motivation to quit and in how strongly self-efficacy predicted motivation to quit, with additional day-to-day variability remaining within individuals. The SDs of the random effects were relatively large, consistent with substantial between-person differences in both baseline levels and within-person associations.

**Table 4.** Estimated models predicting motivation to quit at last daily cigarette smoked as a function of change features of within-day trajectories.

	Self-efficacy for cutting down^a^	Attitudes toward smoking^a^
Fixed effects, estimate (95% CI)		
Intercept	3.12^b^ (2.36 to 3.89)	2.61^b^ (1.67 to 3.55)
Range of change^c^	−0.04 (−0.09 to 0.02)	0.00 (−0.06 to 0.07)
Overall level^d^	0.68^b^ (0.64 to 0.72)	−0.10^b^ (−0.14 to −0.06)
Volatility^e^	0.01 (0.00 to 0.03)	0.02 (−0.00 to 0.03)
Overall trend^f^	0.11^b^ (0.10 to 0.12)	0.01 (−0.00 to 0.02)
Acceleration of change^g^	0.09^b^ (0.08 to 0.11)	0.00 (−0.01 to 0.01)
Trajectory shape^h^	0.00 (−0.00 to 0.01)	−0.01^b^ (−0.02 to −0.00)
Time of last survey	−0.04^b^ (−0.05 to −0.03)	−0.03^b^ (−0.05 to −0.02)
Random effects, variance (SD)^i^		
Intercept	1.64 (1.28)	2.48 (1.58)
Slope for overall level	0.21 (0.46)	0.25 (0.50)
Residual	0.40 (0.64)	0.45 (0.67)

a All models included covariates of intervention condition, age, sex at birth, race or ethnicity, and income.

b Statistically significant at α≤.05.

c Range of change: Maximum – Minimum.

d Overall level: mean of scores per person.

e Volatility: rate of intersection with the mean.

f Overall trend: mean of the first derivative.

g Acceleration of change: mean of the second derivative.

h Trajectory shape: mean-centered moderation term of overall trend (mean of first derivative) and acceleration of change (mean of the second derivative).

i Intercept and slope: *r*=–0.11.

**Figure 3.**
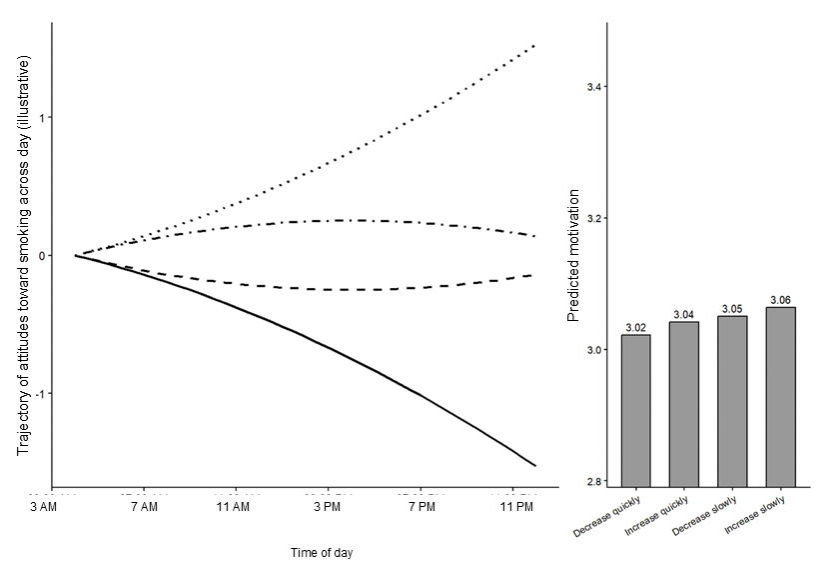
Depiction of moderation effect for how trajectory shape of attitudes toward smoking predicts end-of-day motivation to quit, with illustrative trajectories of predicted attitudes toward smoking trajectories across a day and their corresponding predicted end-of-day motivation to quit smoking. Four hypothetical trajectory types are shown: increasing quickly (positive slope with positive curvature), increasing slowly (positive slope with negative curvature), decreasing slowly (negative slope with positive curvature), and decreasing quickly (negative slope with negative curvature). Labels at the right end of each trajectory display the predicted motivation score from the model. The bar chart to the right summarizes these predicted motivation values, highlighting modest differences across trajectory types.

Table 4 (column 3) shows results of the model testing whether features of within-day trajectories of attitudes toward smoking predicted end-of-day motivation to quit. Overall attitude toward smoking was a significant negative predictor of end-of-day motivation, showing that on days when people had more negative attitudes about smoking, they also felt more motivated to quit at the end of the day. Range of change, volatility, overall trend, and acceleration were not significantly associated with motivation. A small but significant moderation effect emerged for trajectory shape, capturing how the combination of trend and acceleration in attitudes toward smoking related to end-of-day motivation. As illustrated in Figure 3, predicted differences were minimal, with end-of-day motivation values ranging only from 3.02 to 3.06 across a wide variety of predicted trajectories of attitudes toward smoking. Within this narrow range, motivation was slightly higher when attitudes toward smoking became more favorable gradually and slightly lower when they became less favorable more abruptly. The pace of change in attitudes throughout the day mattered minimally for predicting daily motivation to quit. Time of last survey was also significantly associated with motivation, in that motivation to quit was slightly lower when surveys were completed later in the evening. Random effects and their SDs indicated substantial differences between people in their average motivation and some variation in how attitudes predicted motivation, with additional within-person variability across days remaining unexplained.

### Features of Trajectory Predicting Forgoing Cigarette Smoking

The results of the logistic mixed-effects model associating the specific features of within-day trajectories of self-efficacy for cutting down on smoking predicting with the likelihood of forgoing cigarette smoking on that day are shown in Table 5 (column 2). Higher overall levels of self-efficacy were strongly associated with greater odds of forgoing cigarettes, such that a 1-unit increase in average self-efficacy nearly doubled the likelihood of skipping a cigarette. Both overall trend and acceleration of change were also significant predictors: days when self-efficacy moved more consistently upward, and days when those changes became sharper across time, were more likely to include a forgone cigarette. In contrast, the range of change and volatility of self-efficacy were not significant predictors, indicating that the sheer size or noisiness of fluctuations in self-efficacy throughout the day was not associated with the odds of forgoing cigarettes that day. The trajectory shape term was also nonsignificant, suggesting that the combined influence of slope and curvature did not meaningfully add to the prediction of forgoing cigarettes that day beyond their individual effects.

The results of the model testing associations between the specific features of within-day trajectories of attitudes toward smoking predicting the likelihood of forgoing cigarette smoking that day are shown in Table 5 (column 3). More favorable attitudes toward smoking were associated with lower odds of forgoing cigarettes that day. In contrast, greater overall trend and acceleration of change were associated with higher odds of forgoing, suggesting that when attitudes became less favorable and did so more quickly across the day, participants were more likely to have skipped a cigarette at some point that day. Range of change, volatility, and trajectory shape were not significant predictors, indicating that the size, noisiness, and combined slope-curvature pattern of daily fluctuations in attitudes were not significantly associated with forgoing behavior that day.

**Table 5.** Estimated models predicting forgoing cigarettes as a function of change features of within-day trajectories^a^.

	Self-efficacy for cutting down	Attitudes toward smoking
	OR^b^ (95% CI)	OR (95% CI)
Fixed effects		
Intercept	0.03^c^ (0.00-0.19)	0.02^c^ (0.00-0.14)
Range of change^d^	0.95 (0.88-1.02)	1.09^c^ (1.01-1.17)
Overall level^e^	2.20^c^ (1.92-2.52)	0.75^c^ (0.65-0.87)
Volatility^f^	1.03 (0.97-1.11)	0.84^c^ (0.78-0.89)
Overall trend^g^	1.06^c^ (1.01-1.12)	1.12^c^ (1.06-1.17)
Acceleration of change^h^	1.14^c^ (1.08-1.20)	1.00 (0.95-1.05)
Trajectory shape^i^	1.03 (0.99-1.05)	1.01 (0.95-1.05)
Random effects		
Intercept SD	2.84	2.93

a All models included covariates of intervention condition, age, sex at birth, race or ethnicity, and income.

b OR: odds ratio.

c Statistically significant at α≤.05.

d Range of change: Maximum – Minimum.

e Overall level: mean of scores per person.

f Volatility: rate of intersection with the mean.

g Overall trend: mean of the first derivative.

h Acceleration of change: mean of the second derivative.

i Trajectory shape: mean-centered moderation term of overall trend (mean of first derivative) and acceleration of change (mean of the second derivative).

## Discussion

### Principal Findings

The aim of this study was to identify dynamic, within-day trajectories of self-efficacy for cutting down on smoking and attitudes toward smoking and to examine how features of these trajectories relate to end-of-day motivation to quit and the likelihood of forgoing cigarettes. Using a novel trajectory-based approach, we found that both the overall levels and certain dynamic features of these psychological states were associated with cessation-relevant outcomes. Five distinct daily self-efficacy and attitude trajectories were identified, explaining the majority of day-level variance. Across outcomes, overall level emerged as the strongest predictor: higher self-efficacy and less favorable attitudes toward smoking were consistently associated with greater motivation to quit and a higher likelihood of forgoing cigarettes. Importantly, beyond these levels, patterns of change also contributed, such that days characterized by increasing and accelerating self-efficacy were associated with more favorable outcomes. In contrast, other dynamic features (eg, range, volatility, and trajectory shape) were not consistently predictive. These findings highlight how both the level and the pace of change in self-efficacy and smoking attitudes contribute to cessation-relevant outcomes.

Our findings add to the strengthening body of evidence demonstrating that motivation for smoking and smoking cessation are dynamic rather than static traits [25,26,38]. Fluctuations in self-efficacy and attitudes toward smoking likely signify changes in biological states (eg, nicotine withdrawal), physical or social context (eg, being around other smokers and experiencing stress) [25], affective states (eg, positivity, anxiety, and irritability) [39,40], recent smoking-related experiences (eg, failed quit attempt and successful resistance of a craving) [17], and shifting self-control or goals [41]. Consistent with the importance of context, we also found that self-efficacy, attitudes, and motivation to quit were slightly lower when daily surveys were completed later in the evening. This pattern may reflect diurnal changes in self-regulation, including fatigue and cumulative exposure to smoking cues across the day [25,41]. While the causes of these shifts may vary, our findings suggest that the fluctuations themselves carry meaningful information. Patterns in how self-efficacy for cutting down on smoking and attitudes toward smoking change across the day, regardless of their origin, may signal key windows of opportunity for intervention.

Our findings show that changes in key behavioral predictors are systematically linked to motivation and smoking behavior, highlighting just-in-time adaptive interventions (JITAIs) as a promising approach for delivering smoking cessation and reduction interventions at optimal times [27,42]. JITAIs tailor support by continuously monitoring individual-level predictors (eg, attitudes, self-efficacy, cravings, location, and social context) and delivering targeted prompts or techniques (eg, coping strategies, motivational messages, and reminders of quit goals) when an individual is most at risk of smoking or more receptive to treatment effects [42]. By disrupting motivational trajectories that typically lead to lapses, JITAIs can increase the likelihood of sustained abstinence. Interventions have delivered support based on real-time reports of craving, stress, and negative affect [27,37,43]. Related work also suggests that strengthening perceived control and psychological empowerment may support smoking cessation in digital interventions [44]. However, these approaches typically respond to isolated momentary states rather than patterns of change over time.

Our results suggest that incorporating patterning information into decision rules when developing JITAIs, such as rising or declining self-efficacy or shifting attitudes across hours or days, may provide richer information for tailoring intervention delivery. Importantly, these approaches may not require precise or immediate classification into specific trajectory types. Instead, interventions could rely on simpler, continuously updated indicators (eg, current levels and short-term trends) that approximate these patterns, with personalization improving as more data accumulate over time [27,45]. Different patterns may signal distinct intervention needs; for example, periods of low or declining self-efficacy may call for proactive support or coping prompts, whereas high and stable self-efficacy may be better suited to reinforcement or reduced intervention intensity. Similarly, less favorable attitudes toward smoking may present opportunities to reinforce cessation goals, whereas more variable patterns may call for more flexible and responsive support. Further work is needed to determine the minimum data required to reliably detect these patterns and whether they can be identified quickly enough to support real-time intervention delivery. This would allow interventions to leverage early motivation while improving responsiveness to individuals’ patterns over time.

People felt most motivated and were most likely to skip cigarettes on days when their confidence grew and their views of smoking became more negative. These findings build on other evidence showing the significance of dynamics of self-efficacy and attitudes toward smoking for reducing and quitting smoking. For example, momentary decreases in self-efficacy and high volatility of self-efficacy have been found to precede lapses and relapses among those trying to quit smoking [46], and lapses in smoking cessation efforts have been shown to lead to lower overall self-efficacy with more within-day variability [47]. Similarly, more favorable attitudes toward smoking have been linked with lower quit intentions and greater risk of relapse, whereas less favorable or increasingly negative attitudes predict stronger quit motivation and a higher likelihood of abstinence [5,9,11,12]. Tailoring smoking reduction or cessation intervention delivery to moments when self-efficacy is rising and attitudes toward smoking are becoming less favorable may align with periods of greater receptivity, increasing the likelihood that the intervention will be effective [27,45,48], and adding motivational boosters for moments when self-efficacy is declining, fluctuating, or when attitudes are becoming more favorable toward smoking may buffer against self-regulation failures that lead to smoking.

Our findings provided mixed evidence regarding the optimal decision points for targeting attitudes toward smoking as a mechanism for behavior change. On days when attitudes fluctuated more throughout the day, participants tended to report greater motivation to quit by the end of the day; however, they were less likely to have forgone a cigarette that day. As anticipated, individuals with overall more positive attitudes toward smoking were less likely to have forgone cigarette smoking. However, contrary to expectations, a small effect of trajectory shape emerged, indicating that gradual increases in favorable attitudes toward smoking were associated with slightly higher end-of-day motivation, whereas sharper decreases in favorability were associated with slightly lower motivation; however, these differences were minimal. One possible explanation is that the timing of behavior and attitudes may be reversed: because forgoing a cigarette could occur at any point during the day, the decision to forgo may have influenced subsequent attitudes and motivation, as has been shown in prior work [49]. Although several past studies have studied the impact of tobacco advertising on momentary smoking attitudes [50,51], this is among the first of studies to investigate naturally occurring dynamics of smoking attitudes throughout the day. More research is needed to separate attitudes from other related factors such as cravings and mood before we can determine whether changes in attitudes over time are a useful target for optimizing JITAIs.

### Strengths and Limitations

Building on existing evidence of momentary assessments of motivational determinants of smoking behavior [25,26,46,50], a strength of our study was analyses of patterns in the trajectories of self-efficacy and attitudes across a day in a population of people who were mostly unmotivated to quit smoking in the near future. The use of trajectory analyses provided insights into how these determinants tend to change over time and which aspects of change are systematically linked to motivational and behavioral outcomes [34]. The novel combination of cluster-based and parametric analyses provided for both descriptive and inferential insights into the dynamics.

While these strengths offer valuable insights, several limitations should be considered. First, the observational, correlational design of this study with self-reported measures precludes any conclusions about causality or the direction of effects. Experimental and interventional studies with less subjective measures are needed to clarify how changes in self-efficacy and attitudes influence smoking behavior over time. While the data for this study came from a trial to assess responses to different types of cigarette package warning label messages [30,31], we pooled data from across treatment groups because these messages were static and message exposures were not directly assessed, thereby limiting opportunities to evaluate momentary responses to messages may influence the dynamics we studied.

Second, due to the requirements of dynamic analytic methods, we excluded days with fewer event-contingent smoking surveys. On excluded days, participants reported higher self-efficacy for cutting down on smoking, stronger motivation to quit smoking, and higher likelihood of forgoing smoking than on days included in analyses. Consequently, our findings may not generalize to days when participants were most motivated and successful in reducing smoking, potentially biasing the results [52]. Relatedly, few participants in this study reported intending to quit smoking within the next 6 months (32%). However, overall quit intentions were broadly consistent with those observed in the US population: 61% reported intending to quit at some point in the future, 28% were unsure when they intended to quit, and 9% reported no intention to quit. By comparison, in 2022, approximately two-thirds (67.7%) of US adult smokers reported wanting to quit smoking [53]. Our sample contrasts with most EMA studies of smoking cessation, which focus on people who are ready to quit; however, JITAIs appear feasible even for people who are not ready to quit [54,55]—the largest segment of people who smoke and for whom interventions are sorely needed [6].

Third, the exact timing of forgoing smoking events throughout the day was not captured, limiting our ability to examine the temporal relationship between these events and fluctuations in self-efficacy or smoking attitudes. This restricts understanding of whether changes in motivation precede or follow resistance to smoking cues and cravings, a question highlighted as important in prior EMA research [17]. Finally, although we focused on predictors of smoking cessation, our 2-week data collection period was not long enough to capture cessation attempts, which future research should evaluate. Addressing these limitations in future research will be crucial to fully understanding the complex, temporal interplay between motivation, attitudes, and smoking behavior.

### Conclusions

This study extends prior research on smoking-related motivation by moving beyond static assessments of self-efficacy and attitudes to examine how these psychological states change across the course of a day. Through use of a novel trajectory-based approach, this study demonstrated that daily trajectories of self-efficacy for cutting down on smoking and attitudes toward smoking predict both motivation to quit and smoking behavior. Beyond the overall level of these determinants across the day, features of how they change are also systematically linked to motivational and behavioral outcomes. Recognizing and responding to how psychological states evolve, rather than just where they are, can improve the timing, relevance, and effectiveness of digital interventions. It may be that the novel application of dynamically informed approaches to JITAIs could be the key to enhancing the effectiveness of smoking cessation and reduction efforts, thereby reducing the burden that smoking has had on individuals and societies across the world.

## Supplementary material

10.2196/87117 Checklist 1 STROBE checklist.

## Abbreviations

EMA: ecological momentary assessment
JITAI: just-in-time adaptive intervention
OR: odds ratio
